# Immature Immunoglobulin Gene Rearrangements Are Recurrent in B Precursor Adult Acute Lymphoblastic Leukemia Carrying *TP53* Molecular Alterations

**DOI:** 10.3390/genes11090960

**Published:** 2020-08-20

**Authors:** Silvia Salmoiraghi, Roberta Cavagna, Marie Lorena Guinea Montalvo, Greta Ubiali, Manuela Tosi, Barbara Peruta, Tamara Intermesoli, Elena Oldani, Anna Salvi, Chiara Pavoni, Ursula Giussani, Renato Bassan, Alessandro Rambaldi, Orietta Spinelli

**Affiliations:** 1 Hematology and Bone Marrow Transplant Unit, ASST Papa Giovanni XXIII, piazza OMS 1, 24127 Bergamo, Italy; ssalmoiraghi@fondazionefrom.it (S.S.); roberta.cavagna@unimi.it (R.C.); marielorena@gmail.com (M.L.G.M.); gretaubiali@gmail.com (G.U.); mtosi@asst-pg23.it (M.T.); bperuta@asst-pg23.it (B.P.); tintermesoli@asst-pg23.it (T.I.); eoldani@asst-pg23.it (E.O.); asalvi@asst-pg23.it (A.S.); cpavoni@asst-pg23.it (C.P.); arambaldi@asst-pg23.it (A.R.); 2 FROM Research Foundation, ASST Papa Giovanni XXIII, piazza OMS 1, 24127 Bergamo, Italy; 3 DIMET PhD program, Università di Milano Bicocca, 20126 Milan, Italy; 4 Medical Genetics Laboratory, ASST Papa Giovanni XXIII, piazza OMS 1, 24127 Bergamo, Italy; ugiussani@asst-pg23.it; 5 Unità Operativa Complessa di Ematologia, Ospedale dell’Angelo e Ospedale SS. Giovanni e Paolo, via Don Tosatto 147, 30172 Mestre (VE), Italy; renato.bassan@aulss3.veneto.it; 6 Department of Oncology and Hematology, Università degli Studi di Milano, Via S. Sofia, 9/1, 20122 Milan, Italy

**Keywords:** acute lymphoblastic leukemia, *TP53*, immunoglobulin rearrangements

## Abstract

Here, we describe the immunoglobulin and T cell receptor (Ig/TCR) molecular rearrangements identified as a leukemic clone hallmark for minimal residual disease assessment in relation to *TP53* mutational status in 171 Ph-negative Acute Lymphoblastic Leukemia (ALL) adult patients at diagnosis. The presence of a *TP53* alterations, which represents a marker of poor prognosis, was strictly correlated with an immature DH/JH rearrangement of the immunoglobulin receptor (*p* < 0.0001). Furthermore, *TP53*-mutated patients were classified as pro-B ALL more frequently than their wild-type counterpart (46% vs. 25%, *p* = 0.05). Although the reasons for the co-presence of immature Ig rearrangements and *TP53* mutation need to be clarified, this can suggest that the alteration in *TP53* is acquired at an early stage of B-cell maturation or even at the level of pre-leukemic transformation.

## 1. Introduction

Rearrangements of immunoglobulin (Ig) and T-cell receptors (TCR) genes are physiologic mechanisms starting in the early stage of lymphopoiesis and following a hierarchical order in which the DH-JH Ig rearrangement is one of the earliest events. The V, (D), J elements of these genes are juxtaposed in different combinations to generate a diverse set of receptors capable to recognize different antigens. [1,2] Therefore, Ig and TCR rearrangements represent fingerprint-like DNA regions of each distinct lymphoid cell and its descendants, making this peculiar characteristic a suitable marker of clonality when cellular regulatory mechanisms fail. The identification of clonal Ig and TCR rearrangements found clinical application in Minimal Residual Disease (MRD) detection, particularly in Acute Lymphoblastic Leukemia (ALL). [3] Nowadays, it is established that MRD evaluation represents the most important factor for predicting clinical outcome for ALL patients [4,5,6]. Despite the high standardization level reached within European cooperative groups [7], clonality definition and MRD assessment is time-consuming and is not feasible for all the patients, even when it is performed by specialized laboratories. As a consequence, even if conventional risk factors defined at diagnosis still maintain a crucial role, in recent years great efforts were made to identify new additional molecular features useful not only for predicting outcome but also for the most appropriate treatment allocation. The aberrations involving *TP53* gene are detectable in about 10% of adult ALL patients at diagnosis and represent a marker of poor prognosis [8,9,10]. Since the use of intensified treatments could potentially overcome the dismal outcome usually reported in these patients [11], the identification of *TP53* mutations at diagnosis is now mandatory.

Here, we present a study conducted on 171 adult Philadelphia negative-ALL patients in which we analyzed the Ig/TCR molecular rearrangements identified as a leukemic clone hallmark in relation to *TP53* mutational status. All the patients were enrolled in the NILG-ALL 09/00 clinical trial, based on the evaluation of MRD as a decisional tool for the post-consolidation program [5].

## 2. Patients and Methods

We analyzed the DNA samples obtained from the bone marrow or peripheral blood at diagnosis containing at least 30% of blasts of 171 unselected ALL patients enrolled in the NILG-ALL 09/00 clinical trial (ClinicalTrials.gov identifier: NCT00358072). Our cohort of patients included 114 B precursor ALL negative for t(9;22) or BCR/ABL1 transcripts and 57 T-lineage adult ALL. DNA was extracted with commercially available kit (Gentra System, Hilden, Germany) from mononuclear cells separated on Ficoll gradient. DNA samples were studied to identify clonal rearrangements amplifying and sequencing the VDJ/VJ regions of immunoglobulin heavy chain (IgH) or the kappa light chain (IgK), and the T-cell receptor (TCR) gamma (G), delta (D) and beta (B) genes by conventional method [5] or by an integrated NGS-based approach. Libraries for NGS were prepared by a custom NimbleGen SeqCap Target Enrichment assay (Roche, Pleasanton, CA, USA) targeting coding V, D and J genes in the Ig/TCR loci for the identification of D-J and V-D-J rearrangements. Libraries were then sequenced on Illumina MiSeq platform (2 × 300 bp). [12] Clone-specific oligonucleotides were designed based on the unique junction region of each single rearrangement and used to perform MRD evaluation experiments by quantitative PCR, as previously described. [5] The *TP53* gene (exons 4–11) was sequenced by 454 Roche Sequencing and data generated by this procedure were analyzed by GS Run Browser and GS Amplicon Variant Analyzer software as previously described. [9] Based on the platform detection limits, we considered only variants with a variant allele fraction (VAF) above 4%, which were checked in dbSNP, COSMIC and IARC databases to investigate their biological significance. The *TP53* mutations identified with an allele burden >20% were also validated by PCR amplification and Sanger sequencing. Moreover, *TP53* copy number analysis was performed in 158 DNA samples by quantitative PCR method using *hTERT* as reference gene. [9] Statistical tests used in the analysis are Chi-squared or Fisher-exact, as appropriate.

## 3. Results and Discussion

The median age of the 171 ALL patients analyzed in this study was 34.6 (range, 15.6–64.8) and male patients were 57%. Hepatosplenomegaly was present in 53% and CNS involvement in 6%. The cytogenetic profile was normal/not adverse in 56%, not evaluable in 26% and adverse in 18%. The complete remission achieved after induction chemotherapy was 91%. Within the *TP53* exons investigated, 16 mutations (single nucleotide variations and small insertion/deletion) were recognized in 14 patients, accounting for the 8% of the cohort. In particular, the incidence of *TP53* alteration was higher in B-ALL than in T-ALL (10% vs. 5%). We previously demonstrated that these molecular aberrations were associated with the presence of one copy of *TP53* gene and increasing age. Moreover, all patients carrying a *TP53* alteration reached complete remission (CR) after induction therapy but 13 out of 14 relapsed in a short time (within 15 months from CR achievement), showing that the relapse rate was significantly higher in *TP53* mutated than in wild-type subjects. By univariate and multivariate analysis [9], the overall survival was strikingly affected by age, WBC at diagnosis >30 × 10^9^/L and CNS involvement. In addition, patients with a mutated *TP53* gene showed a Leukemia Free Survival (LFS) and Overall Survival (OS) which was dramatically shorter than for wild-type patients, as previously described. [9] The identification of Ig/TCR clonal rearrangements for MRD probe design by conventional PCR was unsuccessful in the majority of the *TP53* mutated cases. Therefore, we retrospectively applied an NGS approach [12] that allows the detection of a larger number of molecular rearrangements. Thanks to this method, the identification of Ig/TCR clonal rearrangements was feasible in all *TP53*-positive patients within the available diagnostic samples (13 out of 14). Interestingly, all the B lineage-ALL patients mutated in the *TP53* gene presented a DH/JH clonal rearrangement (10/10), whereas no T-ALL (0/3) with an aberrant *TP53* presented this type of rearrangement (Table 1). 

The presence of a *TP53* alteration was strictly correlated with an incomplete IgH rearrangement, indeed, the 100% of B precursor ALL mutated for *TP53* carried the DH/JH rearrangement, whereas only the 36% of the wild-type B precursor-ALL showed the same types of rearrangement (*p* < 0.0001). The DH/JH locus recombination is the first event that occurs in the IgH genetic recombination mechanism, followed by the recombination of the VH genes with the rearranged DH-JH portion. As previously described, the detection of an incomplete rearrangement denotes an early stage of maturation of the leukemic cells, often associated with a less mature immunophenotype [13]. Conversely, the complete IgH V-D-J rearrangement was mostly associated with Bp-ALL wild type for *TP53* gene (*p* = 0.02).

Considering this, we investigated the surface antigen asset of our molecularly characterized B cell derived-ALL cohort, and we found that all the *TP53*-mutated patients were defined as a B-I or B-II ALL according to European Group for the Immunological Characterization of Leukemias (EGIL) classification [14], which reflects the sequential stages of B-cell maturation. Furthermore, *TP53*-mutated patients were classified as pro-B ALL more frequently than their wild-type counterpart (46% vs. 25%, *p* = 0.05). Unfortunately, the number of T-ALL mutated for *TP53* analyzed in this research was too small to draw any conclusion about the co-occurrence *TP53* mutation and DH/JH rearrangements, which usually involves about 20% of the T-ALL [15]. In addition, 5 of the 10 *TP53* mutated B-ALL patients which presented a DH/JH clonal rearrangement were evaluable for MRD assessment in at least two consecutive timepoints collected within the NILG protocol (Table 2). At least one of the samples evaluated for each of these patients proved positive for leukemia persistence, suggesting a linkage between *TP53* mutation and MRD persistence. As previously specified, all these patients went toward a clinical relapse.

## 4. Conclusions

In conclusion, although in this study the number of B precursor ALL cases characterized by both the presence of a mutation in the *TP53* gene and an incomplete Ig rearrangement was relatively small, these events were very strictly associated. This observation suggests that the presence of *TP53* mutation should be investigated in all B-precursor ALL at diagnosis in parallel with the search for specific incomplete DH/JH rearrangements for MRD evaluation. At this time, the reasons for the co-presence of *TP53* mutation and this type of incomplete DH/JH rearrangement remain to be clarified. Nevertheless, this co-occurrence can suggest that the alteration of *TP53* is acquired at the early stage of the B-cell maturation, or even at the level of pre-leukemic transformation, as already described for acute myeloblastic leukemia. [16] A comprehensive molecular characterization, now feasible by ad hoc designed NGS panels, allows the identification of *TP53* gene mutations and other prognostically significant genes along with the Ig/TCR rearrangements for MRD evaluation. [12] This combined risk stratification based on molecular genetics at diagnosis and MRD evaluation during treatment remains feasible only at referral laboratories. Nonetheless, the clinical readout is so important that each ALL patient should benefit from such a definition of the risk profile.

## Figures and Tables

**Table 1:** Biological and molecular characteristics of *TP53* mutated ALL NA = Not Available.

Patient ID	Phenotype (EGIL)	Cytogenetics	Genetics	Risk Class	CR	Relapse	CR Duration (Months)	Allo SCT (Outcome)	Survival (Months)	DH/JH	Sanger Seq Validation
BG_4205	B-common	Normal	Negative	SR-B	Y	N	71.3+	N	71.3+	NA	NA
BG_8345	B-common	Normal	Negative	SR-B	Y	Y	6.6	N	17.2	DH3-22/JH6; DH1-26/JH4	Y; Y
BG_2873	pro-B	Unknown	Negative	HR-B	Y	Y	13.8	N	17.4	DH6-6/JH6	Y
BG_11584	pro-B	Unknown	*KMT2A-AFF1*	HR-B	Y	Y	14.7	Y, with disease (dead due to persistent disease)	18.6	DH1-26/JH3	Y
BG_9445	B-common	Hypodiploid	Negative	HR-B	Y	Y	10.2	Y, CR2 (Dead due to relapse)	12.9	DH2-2/JH6	Y
BG_5702	pro-B	Hyperdiploid	Negative	HR-B	Y	Y	5.8	N	8.5	DH2-2/JH5	Y
BG_4254	pro-B	Hyperdiploid	Negative	HR-B	Y	Y	5.0	N	16.4	DH3-9/JH6; DH6-6/JH5	Y; Y
BG_10112	pro-B	Normal	*KMT2A-AFF1*	HR-B	Y	Y	1.8	N	3.7	DH6-6/JH4; DH1-7/JH4	Y; Y
BG_11543	B-common	Normal	Negative	SR-B	Y	Y	1.8	N	6.6	DH2-21/JH6	Y
BG_2097	B-common	Complex	Negative	HR-B	Y	Y	3.7	N	7.3	DH4-23/JH2	Y
BG_6490	pro-B	Hyperdiploid	Negative	HR-B	Y	Y	2.5	N	7.5	DH2-21/JH6; DH2-8/JH3	Y; Y
BG_10442	cortical-T	Hyperdiploid	Negative	SR-T	Y	Y	4.6	Y, CR2 (Dead due to relapse)	11.1	NO DH/JH	Y
BG_8142	cortical-T	Hyperdiploid	Negative	SR-T	Y	Y	5.0	N	16.2	NO DH/JH	Y
BG_8646	cortical-T	Complex	Negative	HR-T	Y	Y	4.0	N	10.2	NO DH/JH	N

**Table 2:** MRD evaluation in the 5 *TP53* mutated patients for whom a molecular probe was available.

Patient ID	Phenotype (EGIL)	Available Probe/s	Rearrangement Used for MRD Evaluation	Method for Probe Identification	MRD Evaluation at PRE4	MRD Evaluation at PRE6	MRD Evaluation at PRE8
BG_9445	B-common	1	DH2JH6A	NGS	- (*)	-	+ (10^−3^)
BG_5702	pro-B	1	DH2JH5A	NGS	+ (NQ)	-	+ (NQ)
BG_11543	B-common	1	DD2DD3	Conventional	+ (10^−3^)	+ (10^−3^)	+ (10^−3^)
BG_2097	B-common	1	DH4JH2B	NGS	-	+NQ	+ (10^−1^)
BG_6490	pro-B	2	DH2JH6A	NGS	+NQ	-	-
			DH2JH3A		+NQ	-	-

NA = Not Available, NQ = Not Quantifiable. Reproducible sensitivity was at least 10^−4^. * Inhibition of the tested sample which can alter the MRD evaluation.
